# Anthropometry, food consumption and iron deficiency anemia, among primary school children (6-15 years) in Kenitra city (North-Western Morocco)

**DOI:** 10.11604/pamj.2021.38.374.10008

**Published:** 2021-04-15

**Authors:** Imane Achouri, Youssef Aboussaleh, Rachid Sbaibi, Ahmed Ahami

**Affiliations:** 1 Behavioral Neurosciences and Nutritional Health Unit, Nutrition and Health Laboratory, Department of Biology, Faculty of Sciences, University Ibn Tofail, BP 133 14000 Kenitra, Morocco

**Keywords:** Food consumption, nutritional status, iron deficiency anemia, school children, Kenitra, Morocco

## Abstract

**Introduction:**

the problem of malnutrition among children is a phenomenon associated with a rapid nutrition transition in Morocco and all developing countries. The objective of this study is to evaluate the nutritional status by anthropometry, food consumption and iron deficiency anemia among primary school children aged 6-12 years in Kenitra city (Morocco).

**Methods:**

the survey covered 271 students (55% of boys and 45% girls) aged 6 to 12. Information concerning food consumption patterns, socioeconomic status and other lifestyle factors was obtained using questionnaires and interviews. Anemia was defined when haemoglobin < 11.5 g/dl.

**Results:**

the results showed that, 6.3%, 2.2% and 17.3% children were stunted, thin and overweight respectively. No significant association was found between gender and nutritional status of children. 16.2% children were anemic and food consumption found not to be varied and below recommendations.

**Conclusion:**

the finding in this study showed that overweight and obesity occurred more frequently than the various forms of under nutrition in the population studied. The finding support the urgent need to improve the nutritional status of children by implementing preventive strategy for the problem of malnutrition among school-aged children.

## Introduction

Malnutrition is the most important public health problem that affects many children in the world and in developing countries [1]. It is the cause of at least half of infant mortality worldwide. The undernutrition alone is responsible for over a third of these deaths [2]. The rates of stunting among children under five years of age are also very high in many Southern and Eastern Mediterranean countries: 26.3% in Albania, 14.9% in Algeria, 28.9% in Egypt, and 22.5% in Morocco [3]. Iron deficiency and iron deficiency anemia are considered to be a major form of malnutrition throughout the world, it contributes towards 50% of the approximated 600 million global anemia cases in preschool and school-aged children [4]. The World Health Organization reported that just over 2 billion people are anemic. The prevalence of anemia among students is 25.4% with the highest rate among preschool-aged children (47.4% all over the world and 67.6% in Africa) [5].

Morocco is passing through a rapid changes in diets and lifestyles that have taken place with globalization and urbanization [6]. This change has negative consequences even if access to food is grown and the food is more diverse, the problem of inappropriate diet and chronic diseases related to diet are always increased [7]. In developing countries like Morocco, anthropometry, despite its inherent limitations, remains the most practical tool for assessing the nutritional status of the community [8]. The objective of the present study is to evaluate the nutritional status, by Anthropometry, iron deficiency anemia and food consumption among primary school children aged 6-15 years in Kenitra city (Morocco).

## Methods

**Place and the sample of study:** a cross-sectional descriptive survey was conducted among 271 school children aged (mean=10.75±1.40 years), ranged between 6 and 15 years, in three primary schools located in different zones , in Kenitra city, in the North West of Morocco.

### Anthropometry

**Anthropometric measurements:** the children's height and weight were measured according to the WHO's guideline [9], with minimal clothing and without shoes. Weight was measured to the nearest 0.1 kg with an electronic scale. Children´s height was measured to the nearest 0.1 cm with a wooden stadiometer placed on a flat surface.

**Indices and anthropometric indicators:** the anthropometric indices of the study population were Height-for-Age (H/A) and Body Mass Index-for-Age (BMI), weight, height, and age data were used to calculate z-scores in accordance with the world health organization (WHO) growth references 2007 for 5 - 19 years [9], using the WHO AnthroPlus Software (Version 10.4, 2010) [10].

**The anthropometric indicators** of stunting and thinness were defined as Height-for-Age (HAZ) and Body Mass Index-for-Age (BAZ) Z-scores < -2, respectively, according to the World Health Organization (WHO) [10].

### Food consumption

#### 24-hour dietary recall

The 24-hour dietary recall interviews were conducted in the schools using face-to-face interactive interview. One 24-h dietary recall was collected for each study participant. The participant is first asked for a quick list of foods consumed during the past 24 hours. Then starting with the first food item listed, the interviewer probes for details of the food consumed, including amounts and additives.

**Iron deficiency anemia:** to evaluate iron deficiency anemia we used five blood tests:

The four following blood analysis: haematocrit (Hct) (%), mean corpuscular volume (MCV) (fl), mean corpuscular haemoglobin (MCH) (pg), mean corpuscular haemoglobin concentration (MCHC) (g/dl) and serum iron (µg/dl), were measured by 5-ml blood sample, collected by antecubital venipuncture and drawn into a container with EDTA for red blood cells (RBC) using an automated cell counter (MS9-5S, Osny, France) by trained and experienced laboratory technicians in private laboratory of medical analysis under suitable conditions.

The last test: hemoglobin (g/dl) level is determined by cyanmethemoglobin method using a Drabkin reagent (D5941) containing potassium ferricyanide K3Fe (CN6) and potassium cyanide (KCN). The potassium ferricyanide converts haemoglobin to methemoglobin. The potassium cyanide then combines with the methemoglobin to form the stable cyanmethemoglobin; the colour intensity is measured spectrophotometrically at 540 nm. The cut-off value for the determination of anemia was defined as blood Hb concentration < 12g/dL in children of 12-14 years and < 11.5 g/dL in children aged 6-11 years [11].

**Statistical analysis:** the statistical analysis was performed using the software IBM SPSS Statistics version 21 (Statistical Package for the Social Sciences). The results are given as figures and tables. Logistic regression was used to investigate the relation between haemoglobin and other haematological parameters. P< 0.05 was considered as statistically significant.

## Results

### Anthropometry

The mean Z score of height for age was -0.24±1.17 and BMI for age was 0.14±1.02. The Table 1 shows the anthropometric characteristics of the sample. Table 2 presents the anthropometric indices (Height for age and BMI for age z scores) depending on the age and sex of the children studied. The percentage of the height / age < - 2 for girls and boys is 8.5% and 4.2% respectively. The percentage of the BMI / age <-2 is 2.3% and 5.6% for girls and boys respectively (Table 2). The prevalence of stunting and thinness decreases with age.

**Table 1:** anthropometric characteristics

Parameters	Mean values ± standard deviation	
Weight in Kg	34,26	± 8,66
Height in cm	139,07	± 9,03
Height for age Z-score	- 0,24	± 1,17
BMI for age Z-score	0,14	± 1,02

**Table 2:** anthropometric indices of nutritional status in children, by age and sex

	Height for age			BMI for age			
	<-2 Z	~ -2 Z	P	<-2 Z	> -2 Z; < +1 Z	>+1 Z	P
	Stunting	Normal stature		Thinness	Normal	Overweight and obesity	
**Sex**							
Girls	11 (8.5%)	118 (91.5%)	0,14	2 (1.6%)	106 (82.2%)	21 (16.3%)	0,69
Boys	6 (4.2%)	136 (95.8%)		4 (2.8%)	112 (78.9%)	26 (18.3%)	
Total	17(6.3%)	254 (93.7%)		6 (2.2%)	218 (80.4%)	47 (17.3%)	
**Age (years)**							
≤ 10	13(10.7%)	109 (89.3%)	0,007	6 (4.9%)	96 (78.7%)	20 (16.4%)	0,023
> 10	4 (2.7%)	145 (97.3%)		0	122 (81.9%)	27 (18.1%)	
Total	17(6.3%)	254 (93.7%)		6 (2.2%)	218 (80.4%)	47 (17.3%)	

### Food consumption

#### Daily food consumption

**Figure 1:**
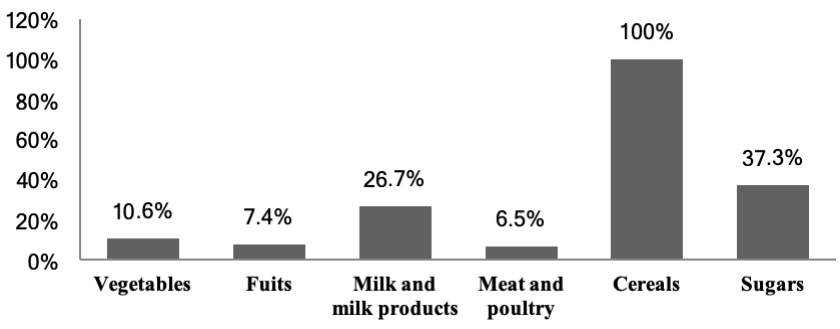
frequencies of food consumption per day

According to Figure 1, fruit and vegetable consumption is low. More than half of the children studied don't consume vegetables daily. Only 10% eat it every day. Fruit consumption is less important than vegetables. Only 7.4% consume fruits daily (Figure 1). The consumption of dairy products is generally low. Only 26% of children drink milk daily. Sugar consumption is very important in the majority of children. It is observed that 37.3% eat sugar every day (Figure 1).

**Figure 2:**
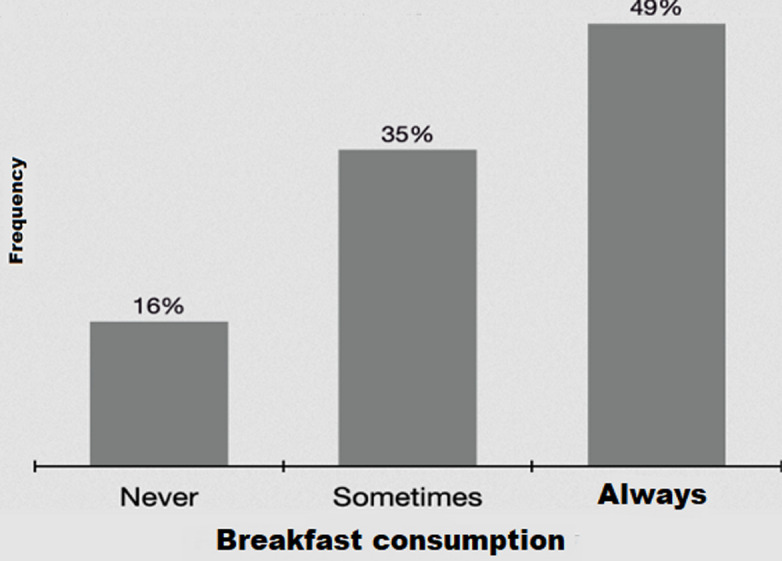
frequency of breakfast consumption

**Breakfast consumption:** Figure 2 shows that 49% of children eat breakfast regularly and 35% skip it at least 2 or 3 times a week.

### Iron deficiency anemia

#### Prevalence and factors influencing Hb levels

In the present study, 16.2% of children were anemic. The mean haemoglobin concentration was 12.45g/dl ± 0.9. Backward-stepwise multiple regression analysis (Table 3) was used to identify the factors influencing haemoglobin levels. Haematocrit (Hct), mean corpuscular volume (MCV), mean corpuscular haemoglobin (MCH), and mean corpuscular haemoglobin concentration (MCHC) were significantly related to haemoglobin levels. The overall F-ratio for all variables was 37.57 and was highly significant (P = 0.000) (Table 3).

**Table 3:** stepwise multiple regression for haemoglobin concentration of school-aged children in Kenitra

Variable	B	ES	β	T	P	95% CI for B
Hct (%)	0.324	0.005	0.759	63.16	0.000	(0.313, 0.334)
MCV (fl)	-0.058	0.009	-0.309	-6.05	0.000	(-0.076, -0.039)
MCH (pg)	0.180	0.027	0.449	6.454	0.000	(0.125, 0.236)
MCHC (g/dl)	0.201	0.024	0.247	8.232	0.000	(0.152, 0.249)
Serum iron (μg/dl)	0.033	0.036	0.011	0.930	0.354	(-0.038, 0.104)

B-Ordinary least-squares regression coefficient; SE-Standard error of B; Beta-standardised β coefficient; T-Observed t value; P-Significance level; CI-confidence interval. Model summary: Multiple R = 0.9; R2 = 0.98; adjusted R2 = 0.964; F-Ratio = 37.57; P = 0.000.

## Discussion

The study sample consists of 271 children whose 55% girls and 45% boys. The average age of children was 10.75 ± 1.40. The finding in this study showed that overweight/obesity occurred more frequently than the various forms of under nutrition (stunting, and thinness) in the population studied. Overall, 6.3%, 2.2% and 17.3% were stunted, thin and overweight respectively. The same result was reported by Aboussaleh *et al*. who found that 7.6% of school children were thin [12]. El Hioui *et al*. reported that 8.9% of school children were stunted stunted in rural region of Kenitra [13], this result is comparable to our finding. In the present study, stunting is higher in girls (8.5%) than in boys (4.2%), but without statistical significance. The relationship between stunting and gender in the previous studies varied. While some similar studies demonstrated a higher prevalence among males [14, 15], others demonstrated a higher prevalence among female [16]. According to some studies conducted in some sub-Saharan African countries, there is important and positive association of stunting with haemoglobin concentration [17, 18]. Another large study in Shaanxi Province, China, found HAZ associated with anemia in school-aged children [19]. The prevalence of stunting and Thinness in the present study decreased with age in both sexes but without statistical significance. This result is comparable to the national survey reported by the Ministry of Health which was decreased from 29% to 23.4% [20]. This may be due to inappropriate child diet, or the onset of puberty which is characterized by a weight and growth fluctuation.

In the present study, the overall prevalence of overweight/obesity was 17.3%, similar finding were seen among school children aged 5 to 12 years in Pakistan which found that 17% were overweight [21]. According to a systematic review carried out by Musaiger *et al*. the prevalence of overweight and obesity among school children in the Eastern Mediterranean region ranged from 7% to 45% between 1990 and 2011 [22]. Another study conducted in Marrakech city in Morocco among 1814 school children (8 - 15 years), reported that the prevalence of overweight and obesity were 8% and 3% respectively based on WHO reference [23]. Thus, this considerable difference in the proportion of overweight and obesity in different regions is may be due to the high prevalence of stunted children that are more vulnerable to be overweight, and cutoff used to estimate overweight and obesity. The percentage of overweight/obesity was higher in boys (18.3%) than in girls (16.3%) but without statistical significance. This finding is consistent with the results reported in India, and Pakistan [21, 24]. There is no significant relationship between nutritional status and gender of the children studied (P > 0.005). Same result was found in a previous studies conducted in Morocco [25] and in India [26] that showed malnutrition also had no sex predilection.

Only 10% of children eat vegetables daily. Fruit consumption is less important than vegetables (7.4%). These rates are below the recommendations of the WHO and FAO who insist on the daily intake of five servings of fruits and vegetables a day to prevent chronic diseases [27]. Consumption of dairy products is generally low. Only 26.7% of children drink milk daily, the majority consume it less than once a week (25%); these rates are lower than the recommendations illustrated by the pyramid of US food guide suggested a daily intake of three servings per day [28]. Furthermore, this study showed that the consumption of meat and poultry is very low (6.5% per day) which are a source of heme iron. There are two forms of dietary iron: non-heme and heme iron. Non-heme iron takes the simplest form of free iron atoms such as ferric (Fe3+) or ferrous (Fe2+) iron. Non-heme iron is obtained from foods such as grains, legumes, fruits and vegetables [29]. It was reported that the consumption of animal source foods was associated with a decreased risk of stunting and underweight, and also the existence of iron deficiency anemia among school age children [30]. Furthermore, a study that was carried out by Dror and Allen, reported that consuming animal source foods not only decreased stunting but also improved other anthropometric indices toward the reduction of morbidity and mortality among undernourished children [31].

The consumption of sugars in the present study was higher (37.3%) than the recommended proportions which generally should not exceed 10% of carbohydrate intake [32]. Excessive intake can cause behavioural problems in children [33]. Breakfast is the most important meal of the day. In school children, breakfast improves concentration and school performance [34]. In our study, breakfast is often skipped by 35% of children and never taken by 16% of them. Similarly, a study conducted in the United States among children aged 6 to 11 years, showed that 26% of them, skipped their breakfast while 22% never eat it [35]. Previous studies have shown that only 19% of children (age range: 6-12 years) skipped their breakfast and 4% never eat it [36]. These rates are lower than our finding. This could be explained by the difference in the study area, the food consumption, and the greater number of children from low socioeconomic status in the sample. Overall, there is an association between breakfast consumption and body weight. Some studies [37-39] reported that breakfast skippers are more likely to have the body mass index (BMI) or weight higher than children who consumed breakfast regularly. A high significance between haemoglobin and serum iron was observed according to our correlation test, this result can demonstrate that iron status was to be a major determinant of haemoglobin and hence anemia, similar result was reported by El Hioui *et al*. [40]. Anemia was detected in 16.2% of children in the present study, which was slightly higher than the prevalence found by El Hioui *et al*. (12.2%) in rural Kenitra in Morocco [40]. Another study carried out in the same region in 2003 among 377 children aged 6 to 15 years old indicated a prevalence of 35% [41]. This difference in the prevalence of IDA in these regions may be due to differences in study setting, sample size, inappropriate of food choices and practices, and the difference in socioeconomic and cultural development.

## Conclusion

In the present study, a rapid rise in overweight and obesity among Moroccan primary school children was observed, and about 16.2% of children were anemic. The finding support the urgent need to improve the nutritional status of the schoolchildren by implementing preventive strategy for the problem of malnutrition among school-aged children and make their parents aware of the healthy nutritional habits and the body's requirements from the different nutrients.

### What is known about this topic

- In Morocco, malnutrition is widespread public health problem;
- Iron deficiency anemia is one of the most frequent health problems in the world and it's an indicator of both poor nutrition and health problem;
- A varied diet rich in fruits and vegetables is recommended to prevent both deficiencies and chronic diseases linked to nutrition.

### What this study adds

- The evaluation of nutritional status, iron deficiency anemia and food consumption among primary school children in Kenitra city (North-Western Morocco);
- The present study approved the impact of the national iron fortification project among primary school age children in Kenitra city, which is the improvement in the mean rate of haemoglobin and the decrease in the prevalence of anemia;
- Further studies are needed among primary school age children and strategies are necessary to improve the nutritional status and reduce the problem of anemia.
